# Relationships Between Depressive Symptoms, Dietary Inflammatory Potential, and Sarcopenia: Mediation Analyses

**DOI:** 10.3389/fnut.2022.844917

**Published:** 2022-02-17

**Authors:** Guo-Qiang Chen, Gang-Pu Wang, Ying Lian

**Affiliations:** ^1^Department of Health Management and Shandong Engineering Laboratory for Health Management, The First Affiliated Hospital of Shandong First Medical University & Shandong Provincial Qianfoshan Hospital, Jinan, China; ^2^Department of Medical Record Management and Statistics, Shandong Provincial Qianfoshan Hospital, The First Affiliated Hospital of Shandong First Medical University, Jinan, China; ^3^Department of General Surgery, The Fourth People's Hospital of Jinan City, Jinan, China

**Keywords:** mediation analyses, sarcopenia, diet, inflammation, depressive symptoms

## Abstract

**Background:**

Sarcopenia is a major public health problem. Depressive symptoms and dietary inflammatory potential play important roles in the development of sarcopenia. We aimed to disentangle the relationships between depressive symptoms, dietary inflammatory potential, and sarcopenia.

**Methods:**

A total of 6,082 participants from the National Health and Nutrition Examination Survey (NHANES) were included in the analyses. Sarcopenia was defined according to the Foundation for the National Institutes for Health (FNIH) criteria. The Depressive symptoms were assessed using the nine-item Patient Health Questionnaire (PHQ-9). Dietary Inflammatory Index (DII) was calculated based on 24-h dietary recall interview. Two sets of mediation models were constructed separately.

**Results:**

Depressive symptoms and DII were associated with sarcopenia, with odds ratios [ORs] (95% CIs) 2.54 (1.27, 5.13) and 1.17 (1.00, 1.37), respectively. DII score mediated the association of depressive symptoms with low muscle mass, explaining a total of 10.53% of the association (indirect effect = 0.004). Depressive symptoms had a significant mediating effects on the association between DII with low muscle mass, explaining a total of 12.50% of the association (indirect effect = 0.001).

**Conclusions:**

Our findings suggested that both depressive symptoms and dietary inflammatory potential had direct effects, and indirect effects on low muscle mass, handgrip strength, muscle mass, through each other. It provides important insights into integrated nutritional and psychological intervention strategies in preventing sarcopenia.

## Introduction

Sarcopenia, a syndrome characterized by a progressive and general loss of muscle mass and strength, is associated with increased adverse outcomes, such as falls, functional decline, frailty, and mortality (1–3). It occurs commonly as an age-related process, with the prevalence of sarcopenia rising in the era of aging (4). Muscle mass gradually decreases from 30 years old and accelerates gradually as individual ages, accompanied by low muscle strength or poor physical performance (5). A systematic review indicated that sarcopenia often co-occurs with depression (6). Both conditions, separately, are associated with a range of deleterious consequences, whereas the comorbidity can result in particularly worse outcomes (7). Therefore, it is imperative to identify the exact association between depressive symptoms and sarcopenia and explore the potential mechanisms underlying the association.

Existing epidemiological studies have explored the association between depressive symptoms and sarcopenia, with some reporting an increased risk associated with depressive symptoms (8). Regarding the potential mechanisms, depressive symptoms and sarcopenia share common pathophysiological pathways related to neurotrophins, inflammation, and oxidative stress, in which inflammation is one of the most frequently cited mechanisms (9, 10). For example, an increased level of inflammatory cytokines could lead to the neuroinflammation, which contributes to depression (11). Meanwhile, increased inflammatory cytokine levels could trigger the neuroinflammation pathways involved in pathophysiological processes of sarcopenia (12). Evidence is accumulating that both depressive symptoms and inflammation are potentially modifiable status and can be improved through effective strategies (13, 14). Thus, contextualizing chronic inflammation within broader biopsychosocial models of sarcopenia would provide further insights into the development of preventive and therapeutic targets (15).

Levels of inflammation can be modulated by lifestyle behaviors, such as diet. Recent studies have indicated that nutritional assessment and management may have a potential implication in preventing sarcopenia (16). The Dietary Inflammatory Index (DII) was designed to determine the inflammatory potential of the overall diet (17, 18). Moreover, existing studies conducted in older adults have demonstrated that a higher DII score positively correlates with an increased risk of sarcopenia (19). Simultaneously, the inflammatory potential of diet may associate bi-directionally with depressive symptoms. One recent longitudinal study showed that an anti-inflammatory diet is associated with a lower risk of depression (18). In addition, mental health, such as depressive symptoms, has been found to affect the dietary choice or preference, causing more consumption of nutrients related to low diet quality and higher DII (20, 21). Given these findings on the close relationships between depressive symptoms and DII and between DII and sarcopenia, it is reasonable to hypothesize that there may be mediating effects of depressive symptoms and DII on the development of sarcopenia and differential relationships between depressive symptoms and sarcopenia among individuals with different DII level.

Therefore, data from the National Health and Nutrition Examination Survey (NHANES) were used to (1) report the relationships between depressive symptoms, DII, and sarcopenia, (2) explore the mediating effect of DII and depressive symptoms on the development of sarcopenia, and (3) examine the relationships between depressive symptoms and sarcopenia among individuals with different DII level.

## Methods

### Study Sample

The NHANES is a nationally representative survey on the US population aimed to assess health and nutrition status. The periodic cross-sectional surveys were conducted every 2 years using a stratified multistage clustered probability sampling approach by the US National Center for Health Statistics (NCHS). Participants completed questionnaires, underwent a medical examination, and provided fasted blood samples. The details of NHANES are available elsewhere (22). The survey was approved by the NCHS Institutional Review Board (Protocol #2011-17). All informed consents had been obtained from participants (23).

Data from two NHANES cycles 2011–2014 were enrolled in the present study. Participants being under 18 years old and over 60 years old (*n* = 11,586) were excluded. We further excluded participants with missing data on sarcopenia (*n* = 1,757), depressive symptoms (*n* = 341), and DII (*n* = 165). The final analytical sample thus included a total of 6,082 participants. The flow chart of the study sample was shown in Supplementary Figure 1.

### Assessment of Sarcopenia

Sarcopenia was defined as the presence of weakness and low muscle mass according to the Foundation for the National Institutes for Health (FNIH) criteria (24). The appendicular lean mass (ALM) was assessed by dual-energy x-ray absorptiometry (QDR-4500 Hologic Scanner Bedford, MA, USA). ALM was the sum of lean mass from both arms and legs. Low muscle mass was identified as the ALM adjusted for body mass index (BMI) <0.512 for women and 0.789 for men. Handgrip strength was assessed by using a handheld dynamometer. Participants were asked to exert maximum effort three times for each hand, and the highest value measured was used. Relative grip strength was calculated as the highest grip strength divided by BMI. Weakness was defined as the relative grip strength <1.00 for men and 0.56 for women.

### Assessment of Depressive Symptoms

Depressive symptoms were assessed using the nine-item Patient Health Questionnaire (PHQ-9). The PHQ-9 is composed of items related to symptoms of depression, which has been well-validated in previous studies (25). Each item was scored from “0” (not at all) to “3” (nearly every day), and the total score ranged from 0 to 27, with higher scores indicating higher levels of depressive symptoms. A cutoff score of ≥10 was used to define the presence of depressive symptoms.

### Assessment of DII

The DII was computed based on the dietary intake data gathered by 24-h dietary recall. We calculated the DII score for 27 food parameters available, such as carbohydrate, energy, protein, fat, fiber, cholesterol, saturated fatty acids, monounsaturated fatty acids, polyunsaturated fatty acids, β-carotene, vitamins A, B1, B2, B6, B12, C, D, and E, folic acid, iron, magnesium, zinc, selenium, omega-3, and omega-6 polyunsaturated fatty acids, alcohol, and caffeine. The detail of the DII calculation method developed by Shivappa et al. is available elsewhere (17).

### Covariates

The following sociodemographic and health-related characteristics were included in analyses: age, sex, race (non-Hispanic White, Non-Hispanic Black, Mexican American, Other Hispanic, and other race), educational level (below high school, high school and above), marriage status (married/living with partner, widowed/divorced/separated/never married), family poverty income ratio, smoking status (never, current, and former), drinking status (no, yes), physical activity level (low, moderate, and high), BMI status (underweight/normal: ≤24.9 kg/m^2^, overweight/obese: >25 kg/m^2^), diabetes, and hypertension. Diabetes was defined as (1) a self-reported previous diagnosis by healthcare professionals, (2) fasting plasma glucose level of 7.0 mmol/L or higher, (3) HbA1c concentration of 6.5% or higher, or (4) use of glucose-lowering medications (insulin or oral hypoglycemic medications). Hypertension was defined as the average systolic blood pressure ≥140 mmHg or diastolic blood pressure ≥90 mmHg, or use of anti-hypertensive medication.

### Statistical Analyses

**Figure 1:**
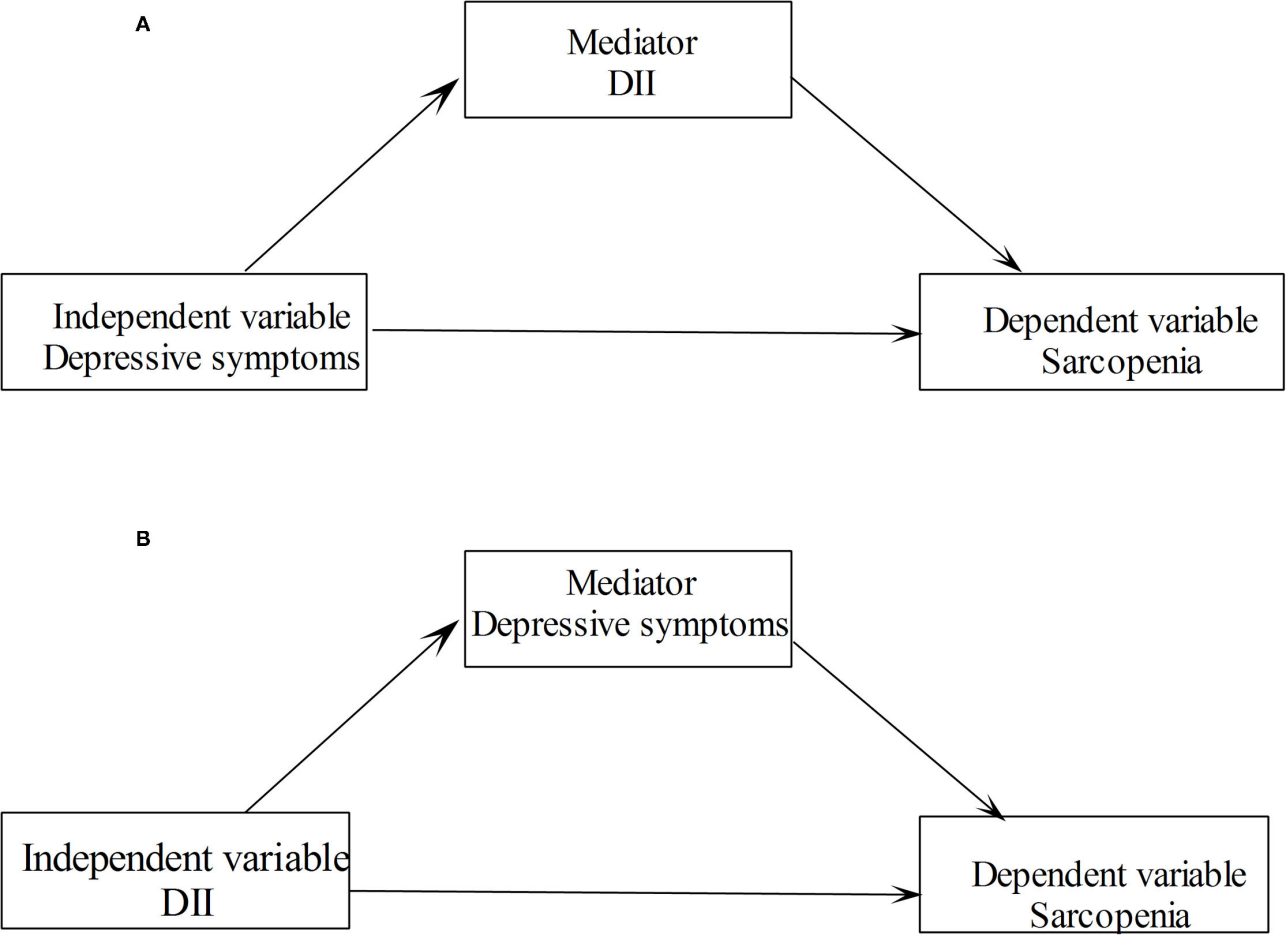
Path diagram of the mediation analysis models.

The characteristics of the study population were analyzed using Student's *t*-tests and Chi-square tests for continuous and categorical variables, respectively. The correlations between DII score and depressive symptoms score with the sarcopenia clinical measures were assessed by the Spearman correlation coefficients. Logistic regression models were used to explore the relationships between depressive symptoms and DII with sarcopenia. Linear regression models were used to explore the relationships between depressive symptoms and DII with the measurements of sarcopenia. For dichotomous outcome variables, the models worked out odds ratios (ORs) and 95% CIs, and for continuous variables, the models worked out βs and 95% CIs. Several confounders, such as age, sex, race, educational level, marriage status, family poverty income ratio, smoking status, drinking status, physical activity level, BMI status, diabetes, and hypertension, were controlled in multivariable regression models. Two sets of mediation models were constructed and analyzed separately. In a mediation model, we included sarcopenia and its measurements as dependent variables, depressive symptoms as the independent variable, and the DII score as the mediating variable (see Figure 1A). We also investigated the mediating effect of depressive symptoms on the association of DII with sarcopenia in another mediation model (see Figure 1B). We examined the association between depressive symptoms and sarcopenia stratified by DII categories. All analyses were conducted using Stata 14.0 software (StataCorp LP, College Station, TX, USA). All values of *p* were two sides with a statistical significance level of 0.05.

## Results

### Descriptive and Correlation Analyses

Of the 6,082 participants, 51.99% were men and 48.01% were women, with a mean age of 37.22 years. The prevalence rate of depressive symptoms was 8.70% and sarcopenia 1.15%. Table 1 shows the characteristics of participants according to depressive symptoms. Participants having depressive symptoms were likely to be older, women, having low education levels, living alone, and low family poverty income ratio. Regarding the health-related variables, lower physical activity, lower handgrip strength, lower muscle mass, sarcopenia, higher DII, and having diabetes and hypertension were significantly associated with depressive symptoms.

**Table 1:** Sample characteristics stratified by depressive symptoms.

**Characteristics**	**Total** ***N* = 6,082**	**Depressive symptoms** ***n* = 529**	**Non-depressive symptoms** ***n* = 5,553**	***P***
Age (years)	37.22 ± 12.52	39.89 ± 12.72	36.96 ± 12.47	<0.01
Sex (%)				<0.01
Male	3,162 (51.99)	197 (37.24)	2,965 (53.39)	
Female	2,920 (48.01)	332 (62.76)	2,588 (46.61)	
Race (%)				<0.01
Non-hispanic white	2,392 (39.33)	247 (46.69)	2,145 (38.63)	
Non-hispanic black	1400 (23.02)	120 (22.68)	1,280 (23.05)	
Mexican-American	779 (12.80)	49 (9.26)	730 (13.14)	
Other hispanic	541 (8.90)	59 (11.15)	482 (8.68)	
Other race	970 (15.95)	54 (10.22)	916 (16.50)	
Educational level (%)				<0.01
Below high school	1,010 (17.10)	145 (27.47)	893 (16.08)	
High school and above	5,042 (82.90)	384 (72.53)	4,660 (83.92)	
Marriage status (%)				<0.01
Married/living with partner	3,487 (57.34)	230 (43.43)	3,260 (58.70)	
Widowed/divorced/separated/never married	2,595 (42.66)	299 (56.57)	2,293 (41.30)	
Family poverty income ratio (%)				<0.01
≤1	1,569 (25.80)	208 (39.24)	1,361 (24.51)	
1–1.84	1,340 (22.03)	156 (29.58)	1,183 (21.31)	
≥1.85	3,173 (52.17)	165 (31.18)	3,009 (54.18)	
Smoking status (%)				<0.01
Never	3,672 (60.38)	210 (39.77)	3,463 (62.38)	
Current	984 (16.18)	95 (17.93)	889 (16.00)	
Former	1,426 (23.44)	224 (42.30)	1,201 (21.62)	
Drinking status (%)				0.07
No	4,556 (74.92)	413 (78.14)	4,143 (74.61)	
Yes	1,526 (25.08)	116 (21.86)	1,410 (25.39)	
Physical activity level (%)				<0.01
Low	2,549 (41.91)	291 (55.13)	2,257 (40.65)	
Moderate	1,905 (31.33)	139 (26.24)	1,767 (31.81)	
High	1,628 (26.76)	99 (18.63)	1,529 (27.54)	
BMI status				<0.01
Underweight/normal	2,044 (33.61)	147 (27.79)	1,897 (34.16)	
Overweight/obese	4,038 (66.39)	382 (72.21)	3,656 (65.84)	
Hypertension (%)	640 (10.53)	73 (13.80)	567 (10.21)	0.01
Diabetes (%)	593 (9.75)	87 (16.45)	506 (9.16)	<0.01
Muscle mass	0.82 ± 0.21	0.74 ± 0.20	0.83 ± 0.21	<0.01
Handgrip strength	1.46 ± 0.49	1.27 ± 0.48	1.48 ± 0.49	<0.01
Sarcopenia (%)	70 (1.15)	16 (3.02)	54 (0.97)	<0.01
Low muscle mass (%)	536 (8.81)	74 (13.99)	462 (8.32)	<0.01
Weakness (%)	133 (2.19)	26 (4.91)	107 (1.93)	<0.01
DII score	0.70 ± 1.89	1.42 ± 1.68	0.63 ± 1.90	<0.01

Table 2 presents the correlation coefficients between each pair of variables, such as sarcopenia measurements, depressive symptoms scores, and DII. Depressive symptoms scores were negatively correlated with the muscle mass (*r* = −0.14, *p* < 0.05) and handgrip strength (*r* = −0.17, *p* < 0.05). DII was negatively correlated with the muscle mass (*r* = −0.23, *p* < 0.05) and handgrip strength (*r* = −0.21, *p* < 0.05). While, depressive symptom scores were positively correlated with DII (*r* = 0.11, *p* < 0.05).

**Table 2:** Correlations between variables.

**Variables**	**Depressive symptoms scores**	**DII score**	**Muscle mass**	**Handgrip strength**
Depressive symptoms scores	1			
DII score	0.11^*^	1		
Muscle mass	−0.14^*^	−0.23^*^	1	
Handgrip strength	−0.17^*^	−0.21^*^	0.82^*^	1

* *p < 0.05*.

### Relationships Between Depressive Symptoms, DII, and Sarcopenia

After adjustments for potential confounders, depressive symptoms were significantly associated with sarcopenia (OR = 2.54, 95% CI = 1.27, 5.13) and measurements of sarcopenia, such as weakness (OR = 1.89, 95% CI = 1.11, 3.21), low muscle mass (OR = 1.43, 95% CI = 1.05, 1.94), handgrip strength (β = −0.05, 95% CI = −0.08, −0.02), and muscle mass (β = −0.02, 95% CI = −0.03, −0.01). Furthermore, DII was also associated with sarcopenia (OR = 1.17, 95% CI = 1.00, 1.37) and the measurements of sarcopenia. More details are shown in Table 3.

**Table 3:** Relationships between depressive symptoms, DII, and sarcopenia.

**Variables**	**Crude model**		**Adjusted model**	
	**Depressive symptoms**	**DII score**	**Depressive symptoms**	**DII score**
Sarcopenia OR (95%CI)	2.94 (1.66,5.21)^*^	1.11(0.97,1.27)	2.54 (1.27,5.13)^*^	1.17(1.00,1.37)^*^
Weakness OR (95%CI)	2.51 (1.62,3.92)^*^	1.11(0.96,1.17)	1.89 (1.11,3.21)^*^	1.05(0.94,1.18)
Low muscle mass OR (95%CI)	1.66 (1.27,2.16)^*^	1.11(1.06,1.17)^*^	1.43 (1.05,1.94)^*^	1.11(1.05,1.18)^*^
Handgrip strength β (95%CI)	−0.17 (−0.22, −0.13)^*^	−0.05(−0.06, −0.04)^*^	−0.05 (−0.08, −0.02)^*^	−0.01(−0.02, −0.004)^*^
Muscle mass β (95%CI)	−0.07 (−0.09, −0.05)^*^	−0.02(−0.03, −0.02)^*^	−0.02 (−0.03, −0.01)^*^	−0.01(−0.02, −0.005)^*^

*Adjusted model adjusted for age, sex, race, educational level, marriage status, family poverty income ratio, smoking status, drinking status, physical activity level, BMI status, diabetes, and hypertension*.

* *p < 0.05. DII, Dietary Inflammatory Index*.

### Mediation Analyses

In the mediation analyses, the DII score mediated the association between depressive symptoms and low muscle mass, explaining a total of 10.53% of the association (indirect effect = 0.004). Meanwhile, significant mediating effects were established in the handgrip strength and muscle mass. Furthermore, there was a significant mediating effect of depressive symptoms on the association between DII and low muscle mass, explaining a total of 12.50% of the association (indirect effect = 0.001). Meanwhile, significant mediating effects were established for handgrip strength and muscle mass. The results of the mediation analyses are presented in Table 4.

**Table 4:** Mediation pathways among depressive symptoms, DII, and sarcopenia.

**Independent variables**	**Mediator**	**Dependent variable**	**Exposure to mediator**	**Mediator to outcome**	**Direct effect**	**Indirect effect**	**Total effect**	**Proportion Mediated (%)**
Depressive symptoms	DII	Sarcopenia	0.492^*^	0.002	0.015^*^	0.001	0.016^*^	6.25
		Weakness	0.492^*^	0.001	0.019^*^	0.001	0.020^*^	5.00
		Low muscle mass	0.492^*^	0.007^*^	0.034^*^	0.004^*^	0.038^*^	10.53
		Handgrip strength	0.492^*^	−0.006^*^	−0.047^*^	−0.003^*^	−0.050^*^	6.00
		Muscle mass	0.492	−0.006^*^	−0.016^*^	−0.003^*^	−0.019^*^	15.78
DII	Depressive symptoms	Sarcopenia	0.01^*^	0.02^*^	0.002	0.001^*^	0.003	33.33
		Weakness	0.01^*^	0.02^*^	0.002	0.001^*^	0.003	33.33
		Low muscle mass	0.01^*^	0.04^*^	0.007^*^	0.001^*^	0.008^*^	12.50
		Handgrip strength	0.01^*^	−0.05^*^	−0.007^*^	−0.001^*^	−0.008^*^	12.50
		Muscle mass	0.01^*^	−0.02^*^	−0.006^*^	−0.001^*^	−0.007^*^	14.29

*Adjusted for age, sex, race, educational level, marriage status, family poverty income ratio, smoking status, drinking status, physical activity level, BMI status, diabetes, and hypertension*

* *p < 0.05. DII, Dietary Inflammatory Index*.

### Stratified Analyses

In the stratified analyses, depressive symptoms were significantly associated with sarcopenia among participants with the second tertile of DII, with the OR (95% CI) 7.99 (2.50, 25.55). Depressive symptoms were significantly associated with muscle mass, one of the measurements of sarcopenia, among participants with the highest tertile of DII, with the β (95% CI) −0.02 (−0.03, −0.01). The interaction test was not significant (*p* > 0.05). Supplementary Table 1 shows the association between depressive symptoms and sarcopenia stratified by categories of DII.

## Discussion

This study disentangled the complex relationships between depressive symptoms, dietary inflammatory potential, and sarcopenia using a nationally representative sample of the US middle-aged adults. Depressive symptoms and dietary inflammatory potential were found to be significantly associated with sarcopenia and its clinical measurements. Of particular importance, there is a novel finding that DII score significantly mediated the association between depressive symptoms and low muscle mass, and there was a significant mediating effect of depressive symptoms on the association between DII and low muscle mass.

Our findings on the relationships between dietary inflammatory potential, depressive symptoms, and sarcopenia supported and extended those from previous studies. Most of the existing studies indicated that depression may be related to sarcopenia, whereas there are also inconsistent results (26–28). Meanwhile, there was a relative scarcity of research on the relationship between depressive symptoms with sarcopenia conducted in middle-aged adults compared to elderly adults previously (26), most of which had limitations related to the assessment of sarcopenia, such as only using one measurement of sarcopenia (29). Notably, the present study not only extended existing evidence but further highlighted the association between depression and sarcopenia in middle-aged adults, thereby further strengthening the possible application of our observation to a wider range of population. With respect to nutritional factors, though the link between diet and sarcopenia has been more widely studied, mainly focusing on nutritional status and specific nutrients (30), little is known about the role of dietary inflammatory potential, presenting a distinct biological mechanism. Our results suggested that the pro-inflammatory diet, indicated by a higher DII score, was related to a greater odd of sarcopenia and its component of muscle mass. Diet-related inflammation may affect muscle proteolysis and myocyte apoptosis, in turn leading to muscle loss and dysfunction (31). Overall, our findings provide convincing evidence on the hypothesized complex relationships and the necessity of clarifying the mechanisms underlying the relationships.

Mediation analyses suggested that there were significant mediating effects of depressive symptoms on the association between DII and low muscle mass, muscle mass, and hand grip strength. The finding indicated that a higher DII score was related to the higher risk of depressive symptoms, which in turn, led to sarcopenia independent of the direct effects of DII on sarcopenia. Similarly, our previous meta-analysis showed that a pro-inflammatory diet was associated with an increased risk of common mental health outcomes, such as depressive symptoms, anxiety, distress, and the association presented in a dose-response manner (32). Potential mechanisms underlying the association between DII and depressive symptoms may include oxidant-antioxidant imbalance and modified gut microbiota composition and activity (33, 34). Furthermore, we also observed that DII significantly mediated the association of depressive symptoms with low muscle mass and handgrip strength. Even though inflammation has often been mentioned as a potential mechanism in the relationship between depressive symptoms and musculoskeletal health, the mediating effect is the first time to be quantified. Likewise, our finding also coincides with previous literature suggesting that a chronic pro-inflammatory signaling has been identified as a key pathway from depression to a range of health outcomes (35, 36). Reportedly, psychological health has consistently influenced one's dietary intake (37). For instance, depression is thought to disrupt the hypothalamic-pituitary-adrenal axis system, which elevates cortisol levels, encouraging increased consumption of energy-dense foods, which influences the dietary quality and the overall inflammatory potential (38). In line with the psycho-neuro-inflammatory theory, the present results improve our understanding the role of dietary and psychological factors in the pathogenesis of sarcopenia (39). In this sense, resolving both factors through efficient nutritional and psychological intervention might have potential to mitigate neuroinflammatory processes and prevent the development of sarcopenia.

Additionally, depressive symptoms were significantly associated with sarcopenia in the subgroup with a higher level of DII score in the stratified analyses. Previous studies suggested the moderating effect of inflammation on the association between depression and adverse health outcomes (40), such as all-cause mortality (41), to some certain degree supporting our findings. Due to the relatively small number of participants with sarcopenia affecting the statistical power, stratified analyses did not demonstrate significant association in individuals with the highest tertile of DII. Further research is necessary to replicate these findings in a larger sample. Although this result warrants further research, the findings suggest that the existence of depressive symptoms, together with a diet-induced inflammatory state, may be associated with higher odds of sarcopenia. Thus, it may be legitimate that integrated utilization of modulating dietary inflammatory potential and addressing depressive symptoms have important public health benefits in the prevention of sarcopenia.

Our study has several important strengths. First, this study included a nationally representative of the general population with a large sample size, which helps to provide convincing support of the hypothesized relationships. Second, the proposed models using mediation and stratified analyses incorporated psychological factors and lifestyle behaviors related to sarcopenia, which enables an insightful understanding of the inter-relationship of these factors with sarcopenia from a comprehensive perspective, thereby facilitating the development of targeted interventions aimed to prevent sarcopenia. Nevertheless, this study has some limitations that need to be mentioned. First, given the cross-sectional design, it is not possible to elucidate the causal associations among dietary inflammatory potential, depressive symptoms, and sarcopenia. Future studies with longitudinal design should seek to clarify the directionality and temporal relationships. Second, although several important confounding variables have been included in the models, there might be unmeasured covariates having a potential confounding effect on the associations. Future studies incorporating these measures are recommended to verify the present conclusion. Third, the mediation effects in our study were generally small. Considering that the etiology of sarcopenia is complex and multifactorial, other biological mechanisms, such as neuroendocrine responses (42, 43) and epigenetic changes (44), might also play a role in the pathophysiology of sarcopenia. It would be important to understand how these different biological processes influence each other. Moreover, future research is needed to incorporate other intermediate mechanisms in the biopsychosocial model of sarcopenia.

## Conclusions

From a practical point of view, the findings of the present study during middle-aged adults may have significant clinical and public health implications. The observed associations provide a useful perspective for prevention and management of sarcopenia as it implies that sarcopenia is not only an age-related problem, but also one associated with a range of long-term conditions even early in mid-life. Comprehensive psychological and behavioral interventions, such as promoting an anti-inflammatory diet and improving mental health, have the potential for prevention and intervention of sarcopenia at earlier stages of life.

In summary, as hypothesized, depressive symptoms, and dietary inflammatory potential were found to be significantly associated with sarcopenia and its individual component low muscle mass. Specifically, both depressive symptoms and dietary inflammatory potential had significant direct effects, and indirect (mediation) effects on low muscle mass, handgrip strength, muscle mass, through each other in the adjusted mediation analyses. Therefore, integrated intervention strategies, such as promoting an anti-inflammatory diet and improving mental health, are suggested in the prevention of sarcopenia.

## Data Availability Statement

The raw data supporting the conclusions of this article will be made available by the authors, without undue reservation.

## Ethics Statement

The studies involving human participants were reviewed and approved by the National Center for Health Statistics (NCHS) and approved by the NCHS Institutional Review Board (IRB). The patients/participants provided their written informed consent to participate in this study.

## Author Contributions

YL contributed to study concept, acquisition of data, and participated in critical revision of the manuscript. YL and G-QC participated in the analysis and interpretation of data. G-QC and G-PW contributed to drafting of the manuscript. All authors read and approved the final manuscript.

## Funding

This work was supported by Shandong Provincial Key Research and Development Program (Grant no. 2019GSF108196), Center of China–US Sports Economics and Health Engineering of Shandong (Grant no. SDCA20191013), Academic Promotion Programme of Shandong First Medical University (Grant no. 2019QL013), and Shandong Provincial Soft Science Research Program (Grant no. 2020RKB14163). The funding sources had no role in study design, data analysis and interpretation of data, the writing of the manuscript, or the decision to submit the paper for publication.

## Conflict of Interest

The authors declare that the research was conducted in the absence of any commercial or financial relationships that could be construed as a potential conflict of interest.

## Publisher's Note

All claims expressed in this article are solely those of the authors and do not necessarily represent those of their affiliated organizations, or those of the publisher, the editors and the reviewers. Any product that may be evaluated in this article, or claim that may be made by its manufacturer, is not guaranteed or endorsed by the publisher.

## Supplementary Material

Supplementary Figure 1 Flow chart of the study sample.

Supplementary Table 1 Association between depressive symptoms and sarcopenia stratified by categories of DII.
