# Supplementary figures and images for: Relationships Between Depressive Symptoms, Dietary Inflammatory Potential, and Sarcopenia: Mediation Analyses
Source: Front Nutr. 2022 Feb 17;9:844917. doi: 10.3389/fnut.2022.844917 (PMC8891449; doi:10.3389/fnut.2022.844917)

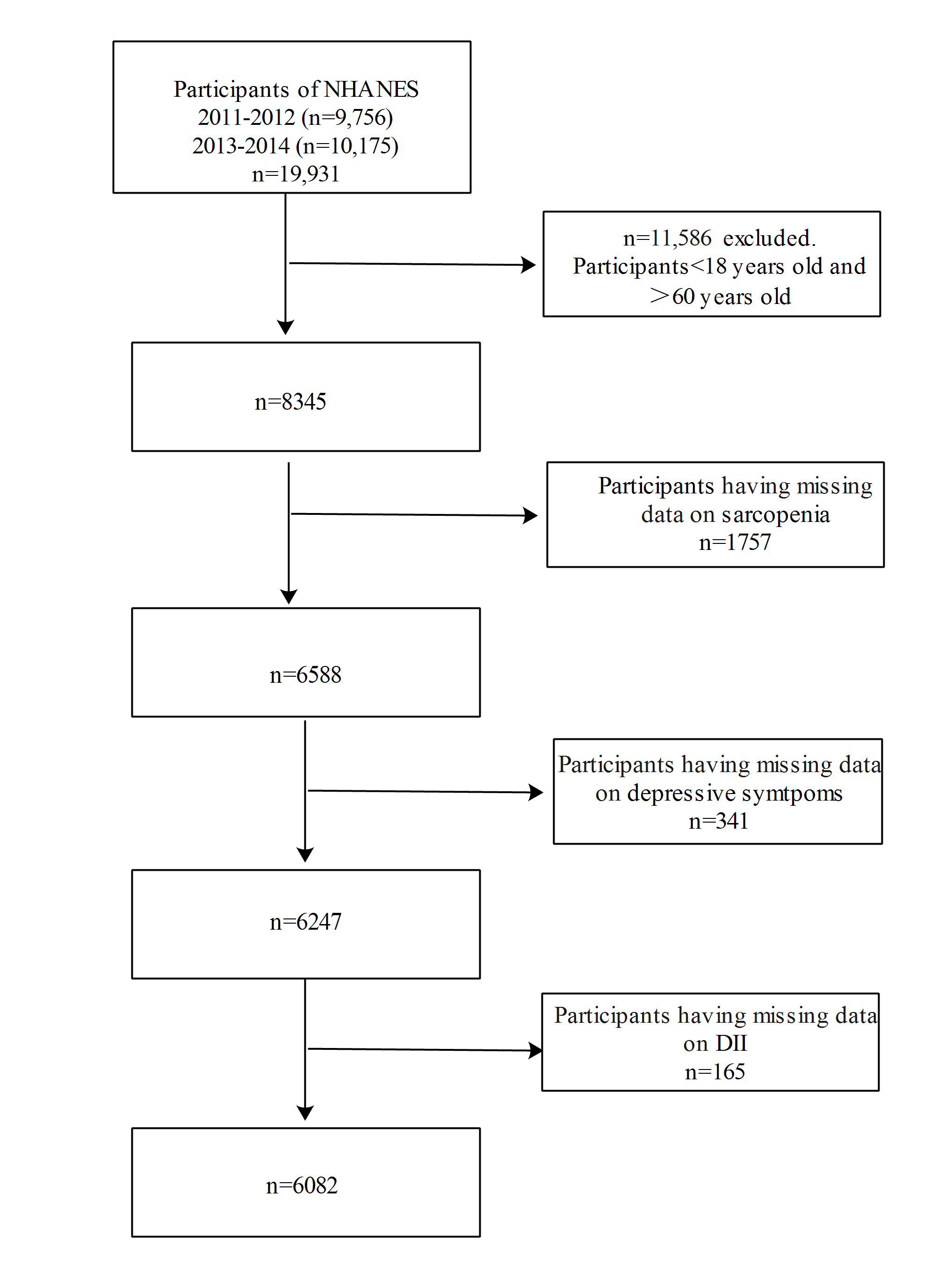

Supplement: Supplementary Figure 1 — Flow chart of the study sample. [file Image_1.TIF]
