# Supplementary material for: Relationships Between Depressive Symptoms, Dietary Inflammatory Potential, and Sarcopenia: Mediation Analyses
Source: Front Nutr. 2022 Feb 17;9:844917. doi: 10.3389/fnut.2022.844917 (PMC8891449; doi:10.3389/fnut.2022.844917)
Supplement: Supplementary Table 1 — Association between depressive symptoms and sarcopenia stratified by categories of DII. [file Table_1.DOCX]

Supplementary Table 1 Association between depressive symptoms and sarcopenia stratified by categories of DII

| Variables | Crude model | Adjusted model |
| --- | --- | --- |
| Tertile 1 of DII |  |  |
| Sarcopenia OR (95%CI) | 2.73 (0.61, 12.18) | 3.08 (0.61, 15.47) |
| Weakness OR (95%CI) | 2.86 (1.09, 7.49) * | 2.14 (0.73, 6.26) |
| Low muscle mass OR (95%CI) | 2.23 (1.23, 4.04) * | 2.02 (0.99, 3.99) |
| Handgrip strength β (95%CI) | -0.09 (-0.13, -0.05) * | -0.08 (-0.15, -0.02) * |
| Muscle mass β (95%CI) | -0.24 (-0.34, -0.14) * | -0.03 (-0.06, -0.01) |
| Tertile 2 of DII |  |  |
| Sarcopenia OR (95%CI) | 4.76 (2.04, 11.10) * | 7.99 (2.50, 25.55) * |
| Weakness OR (95%CI) | 3.71 (1.84, 7.51) * | 3.89 (1.58, 9.61) * |
| Low muscle mass OR (95%CI) | 1.56 (0.97, 2.51) | 1.59 (0.91, 2.77) |
| Handgrip strength β (95%CI) | -0.21 (-0.29, -0.14) * | -0.08 (-0.13, -0.03) * |
| Muscle mass β (95%CI) | -0.07 (-0.10, -0.04) * | -0.01 (-0.03, 0.01) |
| Tertile 3 of DII |  |  |
| Sarcopenia OR (95%CI) | 1.98 (0.79, 4.93) | 0.96 (0.30, 3.13) |
| Weakness OR (95%CI) | 1.59 (1.08, 2.32) * | 1.06 (0.44, 2.59) |
| Low muscle mass OR (95%CI) | 1.78 (0.88, 3.60) | 1.21 (0.77, 1.89) |
| Handgrip strength β (95%CI) | -0.12 (-0.19, -0.06) * | -0.01 (-0.06, 0.03) |
| Muscle mass β (95%CI) | -0.06 (-0.08, -0.03) * | -0.02 (-0.03, -0.01) * |

Note: Adjusted model adjusted for age, sex, race, educational level, marriage status, family poverty income ratio, smoking status, drinking status, physical activity level, BMI status, diabetes, and hypertension. * *P* < 0.05
